# Complete chloroplast genome sequence and phylogenetic analysis of *Magnolia pilocarpa*, a highly ornamental species endemic in central China

**DOI:** 10.1080/23802359.2020.1714511

**Published:** 2020-01-20

**Authors:** Jiaojun Yu, Hongjin Dong, Jun Xiang, Yunli Xiao, Yuanping Fang

**Affiliations:** a Hubei Key Laboratory of Economic Forest Germplasm Improvement and Resources Comprehensive Utilization, Huanggang Normal University, Huanggang, China;; b Hubei Collaborative Innovation Center for the Characteristic Resources Exploitation of Dabie Mountains, Huanggang, China

**Keywords:** *Magnolia pilocarpa*, complete chloroplast genome, Magnoliaceae, phylogeny

## Abstract

*Magnolia pilocarpa* Z. Z. Zhao et Z. W. Xie is a species with high horticultural and medicinal value, which found only in Dabie Mountain of central China. In this study, we sequenced, assembled and annotated the complete chloroplast (cp) genome of *M. pilocarpa*. The length of *M. pilocarpa* complete cp genome was 160,104 bp, with a typical quadripartite structure comprising a pair of inverted repeat regions (IRs; 26,591 bp), a large single copy region (LSC; 88,110 bp) and a small single copy region (SSC; 18,812 bp). The whole cp genome contained 129 unique genes, including 79 protein-coding genes (PCGs), 30 transfer RNA (tRNA) genes and four ribosomal RNA (rRNA) genes. The maximum-likelihood (ML) phylogenetic analysis indicated that *M. pilocarpa* was relatively closed to the *M. denudata*. This chloroplast genome would provide a valuable genetic resource for *M. pilocarpa*.

*Magnolia pilocarpa* Z. Z. Zhao et Z. W. Xie is an endemic species of Magnoliaceae distributed in Dabie Mountains in central China, including Anhui, Henan and Hubei Province. It lives in the sunny slopes from 500 m to 1500 m in altitudes, with obvious sepals in March to April. It was grown as ornamental plant in the adjacent area and the buds were used as the substitutes of the traditional Chinese medicine ‘Xinyi’. Based on the flowering before leaf buds, deciduous vs evergreen (*Magnolia*) and other characteristics, it is separated from *Magnolia* as *Yulania* (Xia et al. 2008).

Genomic DNA was extracted from fresh leaves of *M. pilocarpa* from Wujiashan, Yingshan, Hubei, China (115°48′17.53″E, 31°12′00.92″N, 1450 m; *Dong* et al. *-HGNU-0256*, 2019-04-13; HTGC), the total genomic DNA was isolated according to a modified CTAB method (Doyle 1987). Total genome DNA of *M. pilocarpa* was sequenced by Illumina Hiseq 2500 Sequencing System (Illumina, Hayward, CA) to construct the shotgun library. About 10 Gb pair-end (150 bp) raw short sequence data were obtained and the NOVOPlasty software (Dierckxsens et al. 2017) was used to extract and assemble cp genome. The low-quality sequences were filtered out Using CLC Genomics Workbench v8.0 (CLC Bio, Aarhus, Denmark). The complete chloroplast genome of *M. pilocarpa* was annotated using Geneious ver. 10.1 (http://www.geneious.com [Matthew et al. 2012]) and online program Chloroplast Genome Annotation, Visualization, Analysis, and GenBank Submission (CPGAVAS) (Institute of Medicinal Plant Development, Chinese Academy of Medical Sciences and Peking Union Medical College, Beijing, China) (Liu et al. 2012) and then submitted to GenBank (accession no. MN614308). Finally, a physical map of the genome was drawn by using the online program Organelle Genome DRAW (OGDRAW) (Marc et al. 2013).

The size of chloroplast genome of *M. pilocarpa* is 160,104 bp, which exhibited a typical quadripartite structure including a large single-copy (LSC) region of 88,110 bp and a small single-copy (SSC) region of 18,812 bp separated by a pair identical inverted repeat regions (IRs) of 26,591 bp each. A total of 113 genes were successfully annotated containing 79 protein-coding genes, 30 tRNA genes and four rRNA genes.

To determine the phylogenetic location of *M. pilocarpa* with respect to the other 44 magnoliids and basal angiosperm species with fully sequenced chloroplast genomes, the complete plastome of *M. pilocarpa* was used to reconstruct the phylogenetic relationships. The sequences were initially aligned using MAFFT (Kazutaka and Standley 2013) and then visualized and manually adjusted using BioEdit (Hall 1999). IQ-TREE was used to evaluate and select the best-fitting models of nucleotide sequences (Lam-Tung et al. 2015). Take the plastome of *Amborella trichopoda* (Amborellaceae, Amborellales; AJ506156) as an out-group, a maximum-likelihood analysis was performed with RAxML version 8 program (Alexandros 2014) using 1000 bootstrap. The phylogenetic tree in Figure 1 revealed that *M. pilocarpa* was most closely related to *M. denudate*. All 35 taxa within the Magnoliaceae family were grouped into a clade and clustered into two subclades, one of which was formed by Illicieae, and the other subclade was formed by Magnolieae. The topological structure of which was consistent with the previously-published phylogeny (Gao et al. 2018; Cui et al. 2019; Li et al. 2019). The results will be valuable for the genetic study on genus *Magnolia*.

**Figure 1.**
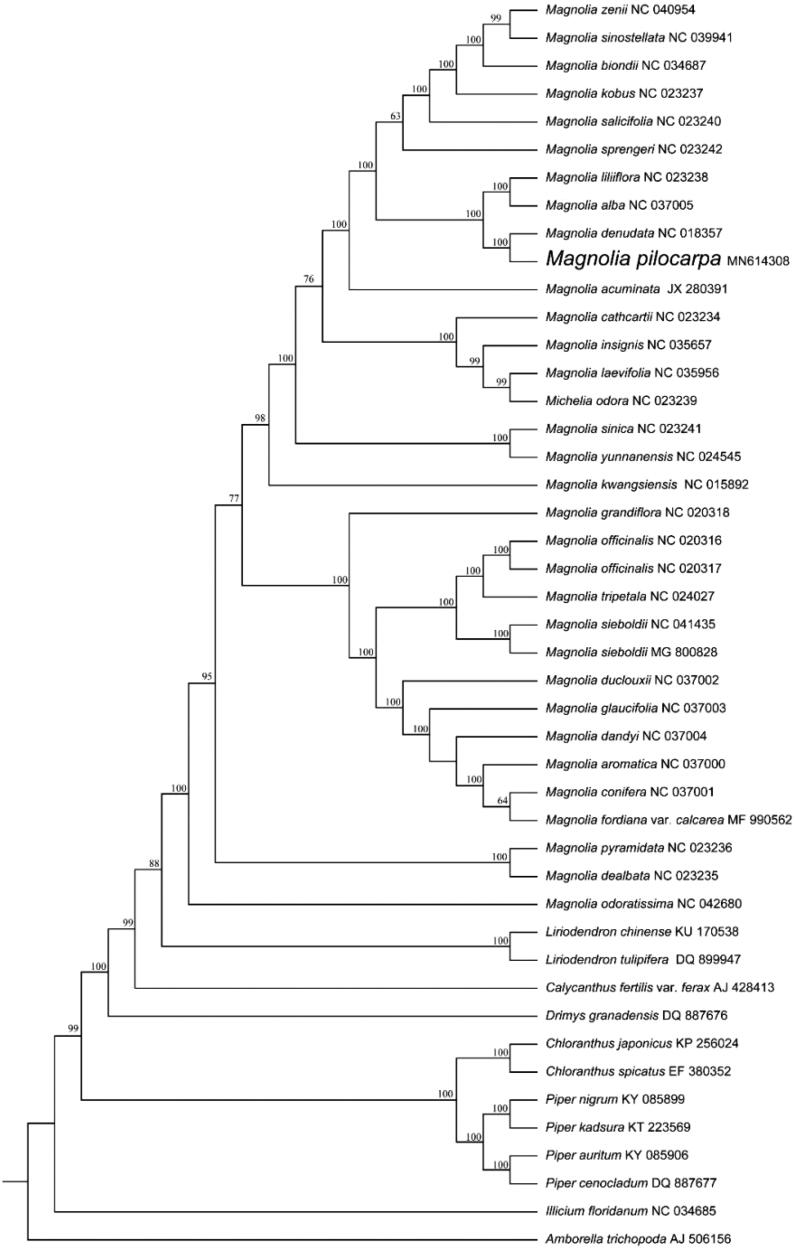
Maximum-likelihood phylogenetic tree for *Magnolia pilocarpa* based on 45 complete chloroplast genomes. The number on each node indicates bootstrap support value.
